# Evaluation of the Optimum Dietary Arachidonic Acid Level and Its Essentiality for Black Seabream (*Acanthopagrus schlegelii*): Based on Growth and Lipid Metabolism

**DOI:** 10.1155/2024/5589032

**Published:** 2024-11-14

**Authors:** Yangguang Bao, Yuedong Shen, Wenli Zhao, Bingqian Yang, Xiaoyi Zhao, Shunshun Tao, Peng Sun, Óscar Monroig, Qicun Zhou, Min Jin

**Affiliations:** ^1^Laboratory of Fish and Shellfish Nutrition, School of Marine Sciences, Ningbo University, Ningbo 315211, China; ^2^Key Laboratory of Aquacultural Biotechnology Ministry of Education, Ningbo University, Ningbo 315211, China; ^3^Key Laboratory of Green Mariculture (Co-construction by Ministry and Province), Ministry of Agriculture and Rural, Ningbo University, Ningbo 315211, China; ^4^Xiangshan Harbor Aquatic Seedling Co. Ltd., Xiangshan County Fisheries Bureau, Ningbo 315702, China; ^5^Instituto de Acuicultura Torre de la Sal (IATS), CSIC, Ribera de Cabanes 12595, Castellon, Spain

**Keywords:** *Acanthopagrus schlegelii*, arachidonic acid, fatty acid composition, growth performance, lipid deposition

## Abstract

The aim of this study was to investigate how dietary arachidonic acid (ARA) level affects growth performance and lipid metabolism in juvenile black seabream (*Acanthopagrus schlegelii*). A feeding trial was conducted for 8 weeks, during which the fish (0.99 ± 0.10 g) were fed six isonitrogenous and isolipidic diets with varying ARA levels of 0.1%, 0.59%, 1.04%, 1.42%, 1.94%, and 2.42%. Fish fed the diet with 1.42% ARA had significantly higher weight gain (WG) and specific growth rate (SGR) than the other groups (*p* < 0.05), except for the ARA1.04. As the ARA level increased, the liver and muscle effectively accumulated *n*−6 polyunsaturated fatty acids (*n*−6 PUFAs; *p* < 0.05). However, eicosapentaenoic acid (EPA), docosahexaenoic acid (DHA) and *n*−3 PUFA contents of liver and muscle significantly decreased by increasing dietary ARA level (*p* < 0.05). Results of liver histology showed dramatically increased vacuolar fat droplets leading to hepatic fat pathological changes in fish fed diets with ARA levels of 1.94% and 2.42% (*p* < 0.05). Serum alanine aminotransferase (ALT) and aspartate aminotransferase (AST) activities increased with increasing dietary ARA level which was accompanied with elevated liver lipid content (*p* < 0.05). Consistently, triglyceride (TG) and nonesterified fatty acid (NEFA) concentrations of serum and liver, and serum cholesterol (CHO) concentration increased (*p* < 0.05). As the level of dietary ARA increased, the indicators of lipid metabolism such as sirtuin 1 (*sirt1*) and peroxisome proliferator-activated receptor *α* (*pparα*) also increased (*p* < 0.05). However, after reaching their peak in ARA1.04 group, the level of these indicators declined (*p* < 0.05). The same trend was observed for the expression of genes related to the downstream pathways. While the mRNA levels of sterol regulatory element–binding protein-1 (*srebp-1*) and its downstream genes were markedly increased with the increase of dietary ARA level (*p* < 0.05). In conclusion, these data suggested that the optimum dietary ARA requirement of *A. schlegelii* is 1.03% of diet based on the WG. The study revealed that a diet containing 1.04% ARA can activate the expression levels of *sirt1* and *pparα* leading to promoted lipolysis. However, dietary ARA levels of ≥1.42% induced lipid accumulation in the liver, as they suppressed the mRNA levels of *sirt1* and *pparα*, while elevating the expression level of genes related to lipogenesis.

## 1. Introduction

The vast majority of freshwater and polar fish can convert dietary *α*-linolenic acid (ALA) to eicosapentaenoic acid (EPA) and docosahexaenoic acid (DHA), and linoleic acid (LA) to arachidonic acid (ARA) [1]. However, most marine fishes are unable to complete this conversion process due to the lack of fatty acid desaturases and elongases [2–4]. It has been well established that ARA, DHA, and EPA play significant physiological and biochemical roles in various organisms [5–7]. These three essential fatty acids (EFAs) are particularly vital for marine fish nutrition, serving as a source of energy and participating in a range of metabolic functions [4, 8–10]. A deficiency in EFAs found in marine fish can detrimentally affect the development and functionality of their nervous and reproductive systems [11]. As a consequence, this can lead to suboptimal growth rates and increased mortality [12]. Although the role of *n*−3 long chain (LC)-polyunsaturated fatty acid (PUFA) in fish nutrition has been extensively studied, the importance of *n*−6 LC-PUFA has also been recognized and studied in recent decades, especially in improving growth and development, reproduction, and stress resistance [13–16]. ARA, a *n*−6 PUFA, is a crucial metabolic hub for unsaturated fatty acids [17]. It not only acts as a precursor for the synthesis of eicosanoids, but also plays significant roles in regulating lipid metabolism and various biological and signaling regulatory processes through its own actions and those of its metabolites [18–21]. Recent studies on ARA nutrition in fish have shown that ARA, as an essential component of phospholipids, is an essential nutrient for growth and survival of fish particularly at juvenile stage [22–25].

Aquatic animals rely mainly on lipids as their primary source of energy [26]. Lipid metabolism is a crucial component of energy metabolism and plays a vital role in providing energy for various physiological processes such as growth, reproduction, and movement in aquatic animals [27]. By efficiently metabolizing lipids, aquatic animals can maintain their energy balance and effectively carry out their life activities [27]. The regulatory role of ARA on lipid metabolic pathways has been well demonstrated in mammals [28]. ARA has been found to have beneficial effects in reducing lipid accumulation in various tissues, and this regulation may be mediated by ARA-derived eicosane compounds, such as prostaglandin e2 (PGE2), which can bind directly to DNA to disrupt gene expression or chromosome structure [28–30]. Evidence suggested that ARA may have the potential as a dietary supplement to prevent obesity and regulate the balance of lipid metabolism in aquatic animals [28, 31]. Dietary ARA inhibited lipid accumulation in adipose tissues and the expression of peroxisome proliferator-activated receptor *γ* (*pparγ*) and fatty acid synthetase (*fas*) in grass carp (*Ctenopharyngodon idellus*) [32]. Likewise, ARA inhibited lipid accumulation through upregulating the expression of peroxisome proliferator-activated receptor *α* (*pparα*) and sterol regulatory element–binding protein-1 (*srebp-1*) genes in the liver of striped bass (*Morone saxatilis*) [31]. Therefore, the relationship between dietary ARA and lipid metabolism is complex and may vary among species, especially in marine fish, accordingly further studies are needed.

*Acanthopagrus schlegelii* is an important euryhaline marine fish, mainly distributed in the western part of the North Pacific Ocean, and is commercially farmed because of its excellent growth performance, strong resistance to adversity, and high economic value [33]. There have been many previous studies on lipid nutrition in black seabream, which focused on the effects of PUFA and *n*−3 LC-PUFA on growth performance, antioxidant capacity, and oxidative stress of black seabream, such as DHA and EPA [33–36]. Nevertheless, the potential role of *n*−6 PUFA, especially ARA, in the regulation of growth performance and lipid accumulation in *A. schlegelii* is not well known. Therefore, the present study was aimed to fill this research gap and investigate the potential regulation of growth performance and hepatic lipid metabolism of *A. schlegelii* by dietary ARA supplementation.

## 2. Materials and Methods

### 2.1. Animal Ethics Statement

The committee on the Ethics of Animal Experiments of Ningbo University (No. SYXK20190005) was established in 2019, but the committee authorities were only for rabbits, mice, and rats, and not aquatic animals. In this study, all juvenile fishes were purchased from commercial farms, and all experimental operations involving animals complied with the requirements of the governing regulation for the use of experimental animals in Zhejiang province (Zhejiang provincial government order No. 263, released on 17 August 2009, effective from 1 0ctober 2010) and were performed according to the Experimental Animal Management Law of China and met the animal operation standards of the industry.

### 2.2. Experimental Diets

The experimental diets were consistent with prior study [37]. Briefly, six isonitrogenous and isolipidic experiment diets were formulated in this study, with 41% protein and 12% lipid. The diets were formulated to contain six levels of ARA (Wuhan Chemstan Biotechnology Co., Ltd., China) and termed as: (1) ARA0.1: 0% ARA, (2) ARA0.59: 0.5% ARA, (3) ARA1.04: 1.0% ARA, (4) ARA1.42: 1.5% ARA, (5) ARA1.94: 2.0% ARA, (6) ARA2.42: 2.5% ARA (Table 1). ARA oil (ARA content, 350 mg/g; DHA content, 64.62 mg/g; EPA content, 0.20 mg/g), palmitic acid, DHA oil, and EPA oil (all from Kenan Biotechnology Co., Ltd., China) were added to the experimental diets to balance the fatty acids content (ARA, DHA, EPA, and DHA/EPA). The feed composition of experimental diets are shown in Table 1, and the complete fatty acid composition and related information of the diets are shown in Table S1. The experimental diets production process was consistent with previous studies [34]. Briefly, according to the proportion and content of nutrients in the feed formula, the corresponding raw materials were ground and mixed in a hammer mill (H-28, South China University of Technology, Guangzhou, China), after passing through a 60-mesh sieve, the obtained ingredients were weighed and thoroughly mixed in a Hobart type blender and cold-extruded pellets produced (F-26, Machine factory of South China University of Technology) with pellet strands cut into uniform sizes (2 mm diameter pellets were prepared; G-250, Machine factory of South China University of Technology). Sinking pellets were dried in an oven for 8–10 h at 45°C to approximately 10% moisture, sealed in vacuum-packed bags and stored at −20°C until used in the feeding trial.

### 2.3. Feeding Trial and Experimental Conditions

*A. schlegelii* juveniles were obtained from a commercial hatchery at Xiangshan Bay (Ningbo, China). Prior to the start of the experiment, black seabream juveniles were acclimated to the experimental facilities and fed a commercial diet (40% protein and 12% crude lipid; Ningbo Tech-Bank Corp., Ningbo, China). A total of 450 black seabream juveniles that were divided randomly into 18 tanks (300 L; 25 fish per tank). Each experimental diet was assigned to three culture tanks. Black seabream juveniles were fed at 8:00 am and 6:00 pm each day. During the feeding trial, the indoor natural ventilation and light was maintained, the farming water was filtered and aerated, and about 40% of the aquarium water was replaced every 2 days to maintain the water quality. During the experimental period, seawater conditions including temperature: 26.2–28.5°C, pH: 7.3–7.8, salinity: 22−23.5 ppt, and dissolved oxygen: 6.35–7.45 mg/L were measured with yellow springs instrument (YSI) Proplus (YSI, Yellow Springs, Ohio, USA).

### 2.4. Sample Collection

After the feeding trial, the fish were fasted for 24 h and then anesthetized using diluted eugenol (4-Allyl-2-methoxyphenol, 10 mg/L), which was dripped into each tank to numb them [38]. The liver samples were collected to analyze enzyme activity and gene expression (pools of three fish per tank, *n* = 3). Additional liver and muscle samples were collected for fatty acid composition analysis and stored at −80°C. Liver from one fish per replicate was sampled for histological analysis. Blood sample was withdrawn using nonheparinized 2-mL syringes from caudal vein of 10 fish per replicate. The blood samples were kept at 4°C overnight, the serum was separated at 4°C after blood stratification, and stored at −80°C for biochemical analysis.

### 2.5. Proximate Composition Analysis

The levels of crude lipid, moisture, and protein in the diets (*n* = 3), as well as the lipid level in the liver or muscle samples (*n* = 3) were determined according to standardized Association of Official Analytical Chemists (AOAC) [39] methods (2006), and the method numbers are AOAC 984.20, AOAC 930.15, and AOAC 992.15, respectively. Moisture content was measured by drying the diet samples to constant weight at 105°C with an oven (DHG-9240A, HuiTai, China). Total lipids were extracted using the Soxtec system HT (Soxtec System HT6, Tecator, Sweden). Meanwhile, protein contents were detected by using the Dumas combustion method with a protein analyzer (FP-528, Leco, USA).

### 2.6. Fatty Acid Composition Analysis

Fatty acid composition of diets, liver, and muscle samples were determined with reference to the previous method and corresponding modification and adjustment [34]. Simply put, the freeze-dried sample (~100 mg) was added to the tubes followed by 3 mL transmethylation solution (99 mL methanol:1 mL H_2_SO_4_:0.025 g butylated hydroxytoluene) was added. Shake well and bathe in 80°C water for 3–4 h. After the water bath, 1 mL n-hexane and 1 mL ultrapure water was added and the mixture was shaken. Then the solution was fully extracted and chromatographed, the filtered supernatant was inhaled into a vial (0.22-μm ultrafiltration membrane; Millipore, MA, USA). The filtered supernatant was quantitatively analyzed using gas chromatography–mass spectrometry (GC-MS; Agilent 7890B-5977A, Agilent Technologies, CA, USA).

### 2.7. Liver Histology

Liver tissue samples were initially fixed in 4% paraformaldehyde solution for histological analysis (Hangzhou Haoke Biotechnology Co., Ltd., Hangzhou, China). After a minimum of 24 h of fixation, the samples underwent gradient dehydration using ethanol ranging from 70% to 100% and were then cleaned and balanced with xylene. Liver specimens were paraffin-embedded, cut into 5-μm sections, and stained with hematoxylin and eosin (H&E). Subsequently, the stained sections were observed under a microscope (Nikon Eclipse CI, Japan), and the relative areas of the liver cells were quantified using Image J software.

### 2.8. Assay of Lipid Metabolism-related Parameters

After collection, the blood samples used to determine the relevant parameters of lipid metabolism were kept at 4°C overnight, the serum was separated at 4°C after blood stratification, and stored at −80°C for biochemical analysis. Samples of liver was homogenized in nine volumes (w/v) of ice-cold physiological saline (0.89%, w/v) before being centrifuged at 956 × *g* at 4°C for 10 min. In accordance with the manufacturer's instructions, the detection of serum indices such as alanine aminotransferase (ALT; C009-1-1, 0~150 U/L), aspartate aminotransferase (AST; C010-1-1, 0~200 U/L), triglyceride (TG; A110-1-1, 0.3~11.4 mmol/L), cholesterol (CHO) (A111-1-1, 0~19.39 mmol/L), nonesterified fatty acid (NEFA; A042-1-1, 0.01~200 mmol/L), high-density lipoprotein-CHO (HDL-C; A112-1-1, 0~5.16 mmol/L), and low-density lipoprotein-CHO (LDL-C; A113-1-1, 0~14 mmol/L) and the contents of TG and NEFA in the liver were all carried out using diagnostic reagent kits (Nanjing Jiancheng Bioengineering Institute, Jiangsu, China).

### 2.9. Quantitative Real-Time Polymerase Chain Reaction (PCR) Analysis

The extraction and reverse transcription of total RNA were based on previous studies [40]. Simply, the concentration of the extracted RNA was measured through the Nanodrop 2000 spectrophotometer (Thermo Fisher Scientific, USA) and subsequently converted into complementary DNA (cDNA) with the PrimeScriptTM RT Reagent Kit (Vazyme, China). The Lightcycler 96 instrument (Roche, Switzerland) was utilized to amplify the RNA and perform quantitative PCR (qPCR), allowing us to assess the expression level of the target genes [40]. The cDNA sequences of the related genes had been uploaded to National Center of Biotechnology Information (NCBI) database and can be found in Table S2.

### 2.10. Statistical and Calculations Analysis

The results are presented as mean ± standard error of mean (SEM) and included the number of biological replicates (*n* value). We compared the average normalized ratio with the control treatment (ARA0.1) expression levels and considered *p* < 0.05 as the significance level to determine the difference among the means. International Business Machines Corporation Statistical Package for the Social Sciences (IBM SPSS) Statistics 20 was used for the statistical analysis, and orthogonal polynomial contrast was used to determine the linear and/or quadratic trends.

The data of liver and muscle fatty acids was analyzed using hierarchical cluster analysis (HCA), by uploading it to a free online program (https://www.bic.ac.cn/BIC/#/). Similarly, after this data was saved in a specific text format, the data was subjected to principal component analysis (PCA) using soft independent modeling of class analogies (SIMCA)—P + software.

## 3. Results

### 3.1. Growth Performance

**Figure 1:**
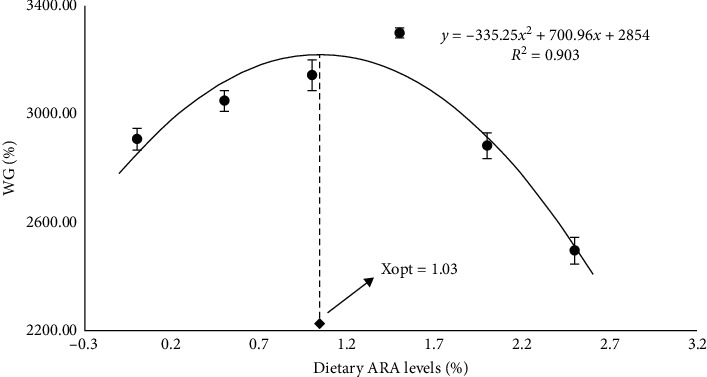
Relationship between WG and dietary ARA (%) levels based on two-slope broken-line regression analysis, where Xopt represents the optimal range of dietary ARA levels for the maximum WG of black seabream (*Acanthopagrus schlegelii*). ARA, arachidonic acid; WG, weight gain.

Results indicated that ARA1.04 and ARA1.42 treatments markedly improved the weight gain (WG) and specific growth rate (SGR) compared to the other groups, except for ARA0.59 group (*p* < 0.05). However, when the ARA levels in the feed exceeded 1.42%, adverse effects were observed on WG and SGR. WG and SGR displayed a quadratic response to dietary ARA level (Table 2). A second-order polynomial regression model based WG showed that the optimum ARA level for *A. schlegelii* is 1.03% of diet (Figure 1).

### 3.2. Liver Fatty Acid Composition

**Figure 2:**
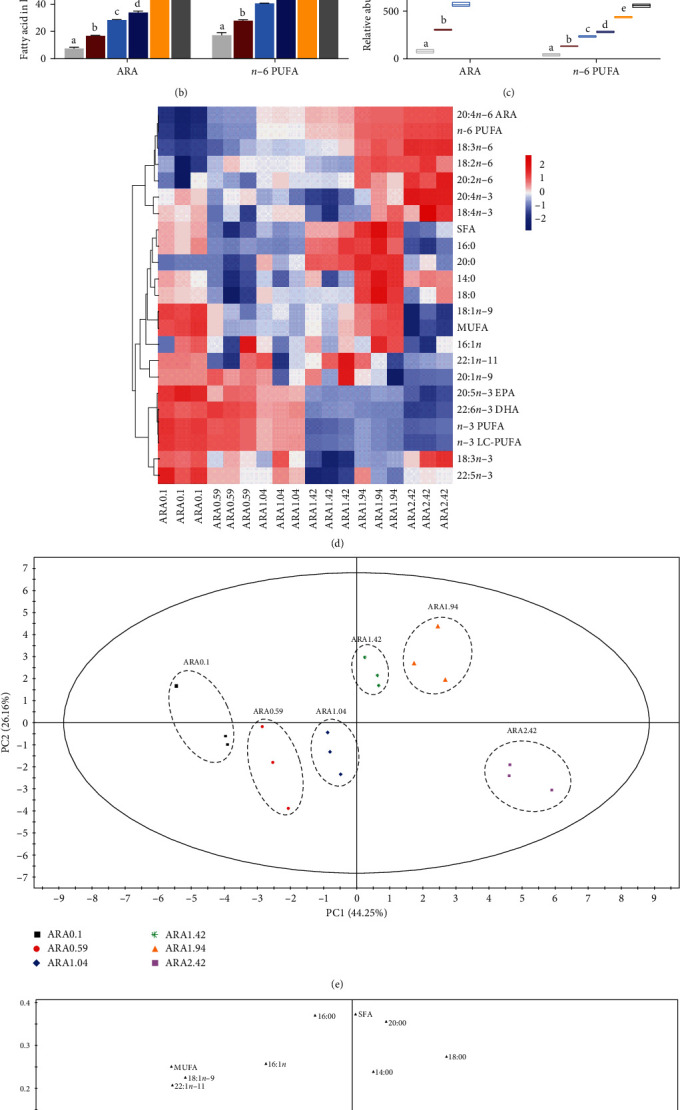
Effects of dietary ARA levels on fatty acid profiles in liver of juvenile black seabream (*Acanthopagrus schlegelii*). Main fatty acid profile in liver (A) and (B), ARA and *n*−6 PUFA relative abundance in liver (C), HCA and heat map visualization of the main fatty acid composition in liver (D), PCA score plot (E), and loading plot (F) results of fatty acids in liver. ARA, arachidonic acid; ARA0.1, 0% ARA; ARA0.59, 0.5% ARA; ARA1.04, 1.0% ARA; ARA1.42, 1.5% ARA; ARA1.94, 2.0% ARA; ARA2.42, 2.5% ARA; DHA, docasahexaenoic acid; EPA, eicosapentaenoic acid; HCA, hierarchical cluster analysis; MUFA, monounsaturated fatty acids; *n*−3 PUFA, *n*−3 polyunsaturated fatty acid; PCA, principal component analysis; SFA, saturated fatty acids. Values are means (*n* = 3), with their standard error represented by vertical bars. Means in the same row with different superscripts are significantly different (*p* < 0.05). ARA relative abundance in liver = (ARA content of experimental liver sample−ARA content of initial liver sample)/ARA content of initial liver × 100; *n*−6 PUFA relative abundance in liver = (*n*−6 PUFA content of experimental liver sample−*n*−6 PUFA content of initial liver sample)/*n*−6 PUFA content of initial liver × 100.

Fatty acid composition can be found in Table S3. As shown in Figure 2, ARA and *n*−6 PUFA contents significantly increased with an increase in dietary ARA level (*p* < 0.05). Conversely, DHA, EPA, *n*−3 PUFA, and *n*−3 LC-PUFA contents noticeably decreased with an increase in dietary ARA level (*p* < 0.05; Figure 2,B). Increasing dietary ARA level led to a significant increasing tendency in the relative abundance of ARA and *n*−6 PUFA in the liver (*p* < 0.05; Figure 2C). Moreover, the heat map indicates the overall effects of different ARA levels in the diet on the fatty acid profile of the liver (Figure 2D). The data was normalized, with high values appearing in red and low values in blue. Fish fed high levels of dietary ARA (1.94%–2.42%) had higher contents of 18:2*n*−6, 18:3*n*−6, 20:2*n*−6, ARA, and *n*−6 PUFA compared to other treatments (*p* < 0.05). Conversely, fish fed low levels of dietary ARA (0.1%–1.04%) had high values of EPA, 22:5*n*−3, DHA, *n*−3 PUPA, and *n*−3 LC-PUFA. The PCA score plot and loading plot indicated the effects of dietary ARA levels on fatty acid profiles in the liver (Figure 2E,F). The first two principal components accounted for 70.41% of the total variation, with principal component (PC)1 contributing 44.25% and PC2 contributing 26.16%. The three replicates for each treatment were clustered together into six clusters, and these clusters were then split. Based on the information obtained from the PCA and the loading plot, it can be observed that ALA, EPA, DHA, *n*−3 PUFA, and *n*−3 LC-PUFA were located on the bottom left side of PC1, which indicates a strong correlation with the ARA0.1, ARA 0.59, and ARA1.04 groups. On the other hand, LA, ARA, and *n*−6 PUFA were distributed on the right side of PC1, showing a powerful correlation with ARA1.94 and ARA2.42 groups.

### 3.3. Muscle Fatty Acid Composition

**Figure 3:**
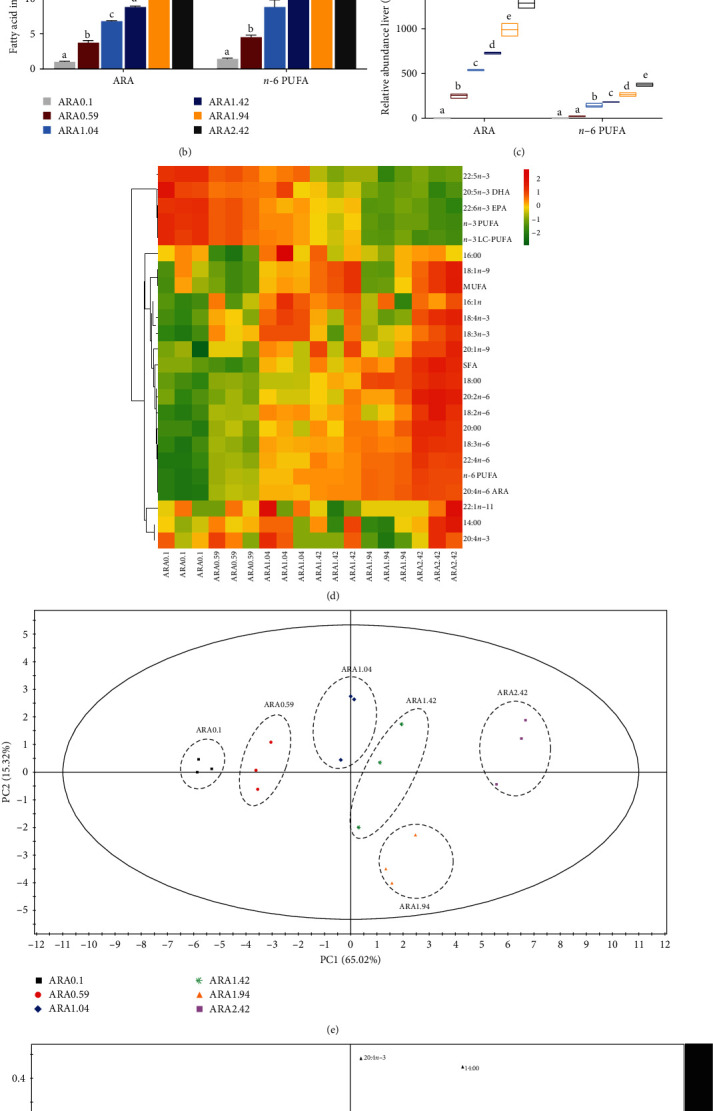
Effects of dietary ARA levels on fatty acid profiles in muscle of juvenile black seabream (*Acanthopagrus schlegelii*). Main fatty acid profile in liver (A) and (B), ARA and *n*−6 PUFA relative abundance in liver (C), HCA and heat map visualization of the main fatty acid composition in liver (D), PCA score plot (E), and loading plot (F) results of fatty acids in liver. ARA, arachidonic acid; ARA0.1, 0% ARA; ARA0.59, 0.5% ARA; ARA1.04, 1.0% ARA; ARA1.42, 1.5% ARA; ARA1.94, 2.0% ARA; ARA2.42, 2.5% ARA; DHA, docasahexaenoic acid; EPA, eicosapentaenoic acid; HCA, hierarchical cluster analysis; *n*−3 PUFA: *n*−3 polyunsaturated fatty acid; PCA, principal component analysis. Values are means (*n* = 3), with their standard error represented by vertical bars. Means in the same row with different superscripts are significantly different (*p* < 0.05). ARA relative abundance in muscle = (ARA content of experimental muscle sample−ARA content of initial muscle sample)/ARA content of initial muscle sample × 100; *n*−6 PUFA relative abundance in muscle = (*n*−6 PUFA content of experimental muscle sample−*n*−6 PUFA content of initial muscle sample)/*n*−6 PUFA content of initial muscle sample × 100.

As shown in Figure 3, major fatty acids concentrations were significantly affected by dietary ARA level. The complete fatty acid composition and related details can be found in Table S4. As expected, increasing dietary ARA level significantly increased the ARA and *n*−6 PUFA contents, while dramatically reduced the EPA, DHA, *n*−3 PUFA, and *n*−3 LC-PUFA contents, which showed a linear correlation (*p* < 0.05; Figure 3A,B). Increasing dietary ARA resulted in an increase in the relative abundance of ARA and *n*−6 PUFA in the muscle (*p* < 0.05; Figure 3C). The heat map visualization (Figure 3D) clearly showed the effects of different levels of ARA on the fatty acid composition profile of the muscle. The data was normalized, with high values shown in red and low values in green. It was evident that fish fed ARA2.42 diet had higher levels of LA, 18:3*n*−6, ARA, 20:2*n*−6, and *n*−6 PUFA. In contrast, the ARA0.1 group exhibited elevated levels of EPA, 22:5*n*−3, DHA, *n*−3 PUFA, and *n*−3 LC-PUFA. Moreover, the PCA and loading plot demonstrated the influence of dietary ARA levels on the distribution of fatty acids in the muscle (Figure 3E,F). The first two principal components accounted for 80.34%, with PC1 accounting for 65.02% and PC2 accounting for 15.32% of the total variance. The three replicates of each treatment are clustered together, for a total of six clusters, and the six clusters were intuitively split. Combining the information from the PCA and the loading plot, it can be observed that EPA, 22:5*n*−3, DHA, *n*−3 PUFA, and *n*−3 LC-PUFA were located on the right side of PC1, indicating a strong correlation with the ARA0.1 and ARA0.59 groups, while LA, 18:3*n*−6, ARA, 20:2*n*−6, and *n*−6 PUFA were distributed on the right side of PC1, showing highly strong correlation with ARA1.94 and ARA2.42 groups.

### 3.4. Liver Histology and Hepatic Lipid Deposition Related Parameters

**Figure 4:**
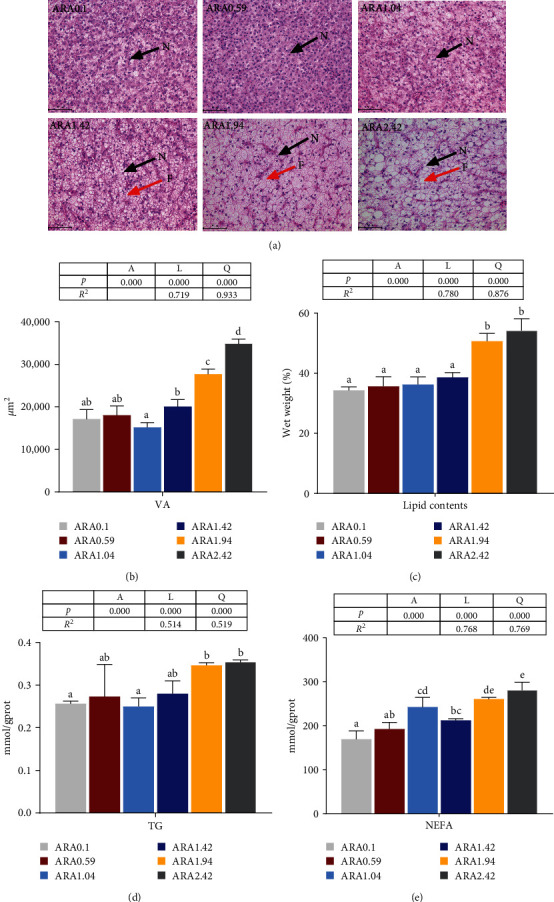
Paraffin section of liver and lipid parameters in juvenile *Acanthopagrus schlegelii* fed with experimental diets. Paraffin section of liver with H&E staining (×400, Bar = 50 μm) (A), VA of liver section (B), the lipid contents of liver (C), contents of TG (D), and NEFA (E) in liver. Values are means (*n* = 3), with their standard errors represented by vertical bars. Means in the same row with different superscript letters are significantly different (*p* < 0.05). F, fat drop; H&E, hematoxylin and eosin; N, cell nucleus; NEFA, nonesterified fatty acid; TG, triglyceride; VA, vacuolar area.

As shown in Figure 4, dietary ARA levels significantly influenced the liver histology and lipid deposition (*p* < 0.05), which were determined by liver cell morphology (Panel A), vacuolar areas (VAs; Panel B), lipid content (Panel C), TG concentration (Panel D), and NEFA levels (Panel E). Dietary ARA levels displayed a significant positive correlation with hepatic lipid deposition parameters (*p* < 0.05). Specifically, the hepatocellular morphology and structure, as well as the nuclei with nucleolus, appeared regular and spherical, with a tightly arranged middle in ARA levels of 0.1%, 0.59%, and 1.04% (*p* < 0.05). However, at ARA levels of ≥1.42% there was a significant increase in the VA (*p* < 0.05). Additionally, excessive dietary ARA levels resulted in a noticeable increase in lipid, TG, and NEFA contents in the liver (*p* < 0.05).

### 3.5. Serum Parameters

**Figure 5:**
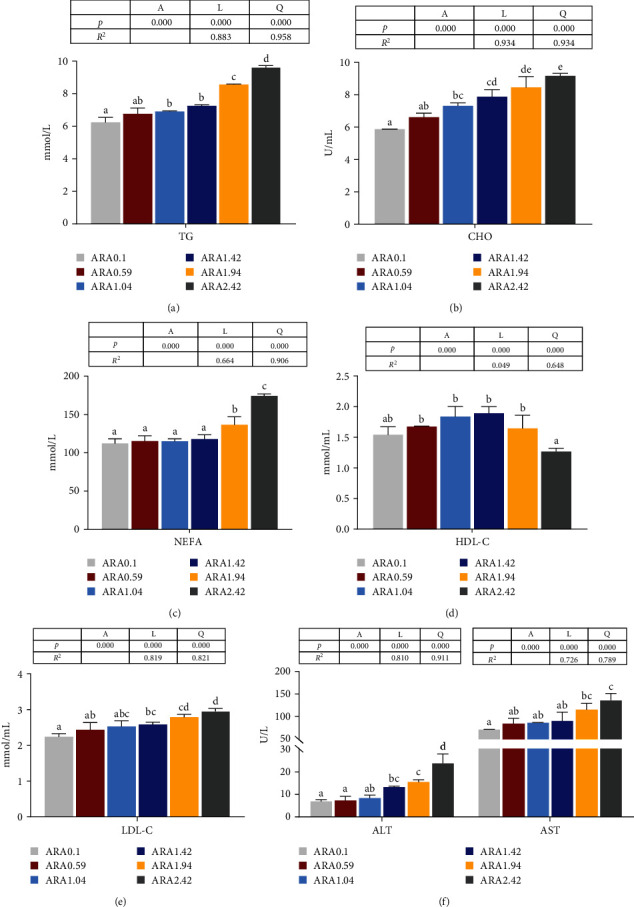
Effects of dietary ARA levels on lipid metabolism related parameters in serum of juvenile black seabream (*Acanthopagrus schlegelii*). The contents of TG (A), CHO (B), NEFA (C), HDL-C (D), LDL-C (E) in serum, and ALT, and AST activities in serum (F). Values are means (*n* = 3), with their standard error represented by vertical bars. Means in the same row with different superscripts are significantly different (*p* < 0.05). ALT, alanine aminotransferase; AST, aspartate aminotransferase; CHO, cholesterol; HDL-C, high-density lipoprotein-CHO; LDL-C, low-density lipoprotein-CHO; NEFA, nonesterified fatty acid; TG, triglyceride.

Dietary ARA levels noticeably influenced serum TG, CHO, NEFA, HDL-C, and LDL-C contents and ALT and AST activities (Figure 5; *p* < 0.05). In particular, with the increase in dietary ARA content, there was a linear and quadratic curve correlations in TG, CHO, NEFA, LDL-C levels, and the activities of ALT and AST, and showed a significant increasing trend (*p* < 0.05). However, the HLD-C content was dramatically reduced in fish fed ARA2.42 diet compared to other treatments (*p* < 0.05), except for dietary ARA0.1 group, which showed significant quadratic curve relationship.

### 3.6. Expression of Lipid Metabolism Genes

**Figure 6:**
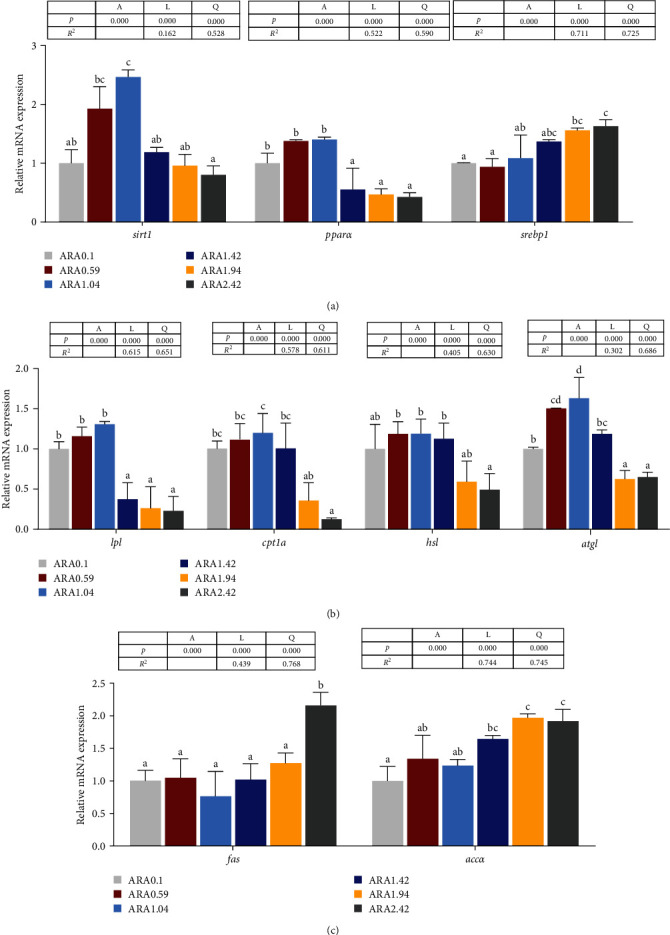
Effects of dietary ARA levels on relative mRNA expression levels of genes involved in lipid metabolism including (A) transcription factors, (B) catabolism, and (C) anabolism in liver of juvenile black seabream (*Acanthopagrus schlegelii*) fed the experimental diets for 8 weeks. The group of ARA0.1 was set as the reference group, and the mRNA expression levels of target genes were normalized relative to the expression of *β-actin*. Values are means (*n* = 3) with their standard error represented by vertical bars. Means in the same row with different superscripts are significantly different (*p* < 0.05). *accα*, acetyl-CoA carboxylase *α*; *atgl*, adipose tricylglyceride lipase; *cpt1a*, carnitine palmitoyltransferase 1A; *fabp*, fatty acid-binding protein; *fas*, fatty acid synthetase; *hsl*, hormone-sensitive lipase; *lpl*, lipoprotein lipase; *pparα*, peroxisome proliferators-activated receptor *α*; *sirt1*, sirtuin 1; *srebp-1*, sterol regulatory element–binding protein-1.

Figure 6 shows the effects of dietary ARA levels on the expression of lipid metabolism related genes in the liver of *A. schlegelii*. The genes related to transcription factor, lipolysis and lipogenesis all displayed significant quadratic curve and/or linear relationships with dietary ARA levels (*p* < 0.05). The mRNA levels of *srebp-1* and *fas*, and acetyl-CoA carboxylase *α* (*accα*) increased significantly with increasing dietary ARA levels (*p* < 0.05). On the other hand, the mRNA levels of sirtuin 1 (*sirt1*), *pparα*, and downstream genes of lipoprotein lipase (*lpl*), carnitine palmitoyltransferase 1A (*cpt1a*), hormone-sensitive lipase (*hsl*), and adipose tricylglyceride lipase (*atgl*) increased initially with increasing dietary ARA levels and peaked in the ARA1.04 treatment, but then significantly decreased with further increase in dietary ARA levels (*p* < 0.05).

## 4. Discussion

As an essential *n*−6 unsaturated fatty acid, ARA has been widely studied in vertebrates including fish [41–43]. Dietary ARA has been shown to improve growth, feed efficiency, and survival in various fish species, making it an important component of fish feed formulation [9, 42, 43]. We conducted a second-order polynomial regression model analysis to determine the optimal dietary ARA requirement for juvenile *A. schlegelii*, which was found to be 1.03%. Similar results have been reported in other aquatic animals such as 0.71%–0.92% for Japanese eel (*Anguilla japonica*) [44], 0.82% for oriental river prawn (*Macrobrachium nipponense*) [45], and 0.22%–0.55% Japanese seabass (*Lateolabrax japonicus*) [46]. We noticed that intake of over 1.42% dietary ARA level negatively affected growth performance. Similar phenomenon was also observed in *Pelteobagrus fulvidraco* [15] and sea cucumber (*Apostichopus japonicus*) [43], suggesting a possible inhibitory effect of excessive dietary ARA intake. It might be attributed to excessive accumulation of *n*−6 LC-PUFA, and subsequently an imbalance of *n*−3/*n*−6 LC-PUFA, resulting in disruption of nutrient metabolism in the organism, especially lipid metabolism, which can lead to suppressed growth performance, which were also confirmed by previous studies [47, 48].

It is widely accepted that tissue fatty acid profile reflects that of the diet [49–51]. As anticipated, dietary ARA supplementation significantly increased the ARA and *n*−6 PUFA levels in tissues, including the liver and muscle. Moreover, there was a positive correlation between the relative proportions of ARA and *n*−6 PUFA in the liver and muscle and the levels of dietary ARA. These findings aligned with a previous study indicating that ARA was effectively deposited in tissues, particularly in the liver and muscle, as dietary ARA supplementation levels increased [21]. Excessive intake of ARA can lead to an imbalance in fatty acid metabolism due to the accumulation of ARA and other *n*−6 PUFA in the liver and muscle [52]. It is noteworthy that when dietary intake of ARA increased, the levels of EPA, DHA, *n*−3 PUFA, and *n*−3 LC-PUFA decreased. This was evident in the heat maps, as well as in the PCA score plot and loading plot. These analyses showed that ARA and *n*−6 PUFA were strongly associated with high levels of dietary ARA, while *n*−3 PUFA was highly correlated with low levels of dietary ARA. It is speculated that there might be a competition between *n*−3 LC-PUFA (such as DHA and EPA) and ARA for phospholipid esterification, which could contribute to decreased DHA and EPA contents in tissues [53]. Together, these results provided additional evidence for the influence of ARA intake on the fatty acid composition in both liver and muscle tissues, that fish intake of high ARA led to increased ARA deposition in tissues and decreased *n*−3 LC-PUFA deposition.

In fish and mammals, liver is considered as a major site for lipid metabolism. It is responsible for the uptake of lipids from diets, transport of lipids throughout the body, secretion of lipids into circulation, as well as the processes of lipogenesis (synthesis of new lipids) and lipolysis (breakdown of stored lipids) [54–56]. However, there have been limited studies exploring the effects of dietary ARA on lipid accumulation in fish and the underlying mechanism. Studies have shown that compression and vacuolization of liver cells are hypothesized to be caused by increased lipid deposition in the livers of fish fed high level ARA diet [28, 56]. According to the study, feeding fish with high levels of ARA (specifically ARA1.94 and ARA2.42) can lead to liver damage. The hepatic histopathological analysis revealed irregular cell shape, obvious lipid vacuole, and noticeable polarization and translocation of the liver nucleus. These findings suggested that overconsumption of ARA in the diet could result in liver damage. Additionally, the high-dose ARA group exhibited an increase in liver fat content, indicating excessive fat accumulation due to excessive addition of ARA in the diet, which aligns with prior observations in Japanese sea bass [28]. ALT and AST are transaminases that play crucial roles in cellular metabolism [57]. These enzymes are crucial indicators of cellular damage response and healthy liver function. Measuring the activity of ALT and AST is crucial in assessing liver function and the extent of cellular damage [57, 58]. The present study revealed that the levels of dietary ARA had a positive correlation with serum ALT and AST activities, which reached their peak at the 2.42% level. These findings suggest that excessive consumption of dietary ARA could cause excessive lipid deposition in the liver, ultimately leading to liver damage.

Herein, the lipid related parameters were assayed in serum and liver to further verify whether high dietary ARA intake can result in lipid accumulation in liver. Results showed significantly higher levels of TGs and CHO in the ARA1.94 and ARA2.42 than those in other treatments. Similar results were found in Japanese eel [44], suggesting that it might be due to ARA being one of the precursors of CHO, and a high dietary ARA level could lead to the accumulation of serum CHO [59]. Besides, NEFA plays a pivotal role in fat metabolism, which is not only an important energy substrate, but also a precursor to the formation of TG storage in adipose tissue, liver, heart, and muscle through esterification [60]. In the present study, the contents of NEFA in serum and liver were significantly increased with the increase of dietary ARA levels. These findings were also confirmed in grass carp indicating that excessive dietary ARA led to the accumulation of excessive TG in the tissues, which contributed to the esterification of excess NEFA during lipolysis, and resulting in systemic metabolic disorders [32, 60]. Additionally, in this study serum HDL-C content increased with increasing dietary ARA levels, while the high concentration of HDL-C was recorded in moderate dietary ARA levels (ARA1.04 and ARA1.42 groups), which could maintain the normal metabolism by clearing excess LDL-C. Combined with the above results, the present study suggested that fish fed with high levels of dietary ARA (1.94% and 2.42%) may cause excess lipid accumulation.

Liver plays a critical role in metabolism of lipids and is an organ that is sensitive to changes in dietary lipid content [61]. This study found that increasing dietary ARA levels led to an increase in the mRNA levels of *srebp-1c* and its downstream genes, including *fas* and *accα*. This indicated that high levels of dietary ARA could activate lipogenesis in the liver by promoting srebp-1c mRNA expression. It was observed that the expression levels of *pparα* and sirt1 followed an opposite trend. Among the diets tested, those containing 1.04% ARA displayed notably elevated expression levels of both genes, compared to those containing 1.42%, 1.94%, and 2.42% ARA. These results indicated that an excess of ARA may hinder the relative mRNA levels of *pparα* and *sirt1*. Along with genes related to lipid synthesis, genes associated with lipolysis can also impact the equilibrium between lipid storage and breakdown within cells [62]. In the current study, the ARA1.04 group exhibited the highest mRNA expression levels of genes related to lipid catabolism including *lpl*, *cpt1α*, *hsl*, and *atgl*. These gene mRNA levels decreased significantly with a further increase in dietary ARA levels. The study indicates a dose-dependent response of genes involved in lipid catabolism to dietary ARA intake. A similar phenomenon was observed in Japanese seabass [28], indicating that fish fed with moderate levels of ARA played an important role in lipolysis and could help restrain excess lipid accumulation. Overall, the results of this study revealed a complex relationship between the levels of ARA in the diet and the genes responsible for regulating hepatic lipid metabolism. The current study suggested that 1.04% ARA in the diet could regulate excessive lipid deposition in the liver by activating *sirt1* and *pparα*. However, excessive intake of ARA in fish could inhibit the expression of *sirt1* and *pparα*, while increasing the expression of *srbep-1c*, aggravating hepatic lipid deposition.

## 5. Conclusion

In brief, the optimal dietary ARA level for juvenile *A. schlegelii* is suggested to be 1.03% of diet based on fish WG. These findings indicated that dietary moderate ARA level (ARA1.04) could avoid excessive lipid deposition in liver by activation of the lipolysis related genes (*sirt1*, *pparα*, *lpl*, *cpt1a*, *hsl*, and *atgl*) to maintain normal physiological functions. However, excessive intake of ARA (1.94%–2.42%) could cause lipid accumulation in the liver by promoting lipogenesis (*srebp-1c*, *fas*, and *accα*) and suppressing lipolysis (*sirt1*, *pparα*, *lpl*, *cpt1a*, *hsl*, and *atgl*). This study comprehensively elucidates the physiological effects of different dietary ARA levels in juvenile black seabream, and provides new insight into the function of ARA on regulating lipid metabolism in farmed marine fish.

## Figures and Tables

**Table 1:** Ingredients and proximate composition of the experimental diets (dry basis, %) [37].

Ingredients	Experimental diets					
	ARA0.1	ARA0.59	ARA1.04	ARA1.42	ARA1.94	ARA2.42
Fish meal	26.00	26.00	26.00	26.00	26.00	26.00
Soy protein concentrate	10.00	10.00	10.00	10.00	10.00	10.00
Soybean meal	20.00	20.00	20.00	20.00	20.00	20.00
Yeast meal	5.00	5.00	5.00	5.00	5.00	5.00
Wheat meal	22.90	22.90	22.90	22.90	22.90	22.90
ARA^a^	0.00	1.40	2.80	4.20	5.60	7.00
DHA^b^	1.30	1.00	0.70	0.40	0.10	0.00
EPA^c^	3.00	3.00	3.00	3.00	3.00	3.00
Palmitic acid	5.70	4.60	3.50	2.40	1.30	1.00
Soybean Lecithin	1.00	1.00	1.00	1.00	1.00	1.00
Vitamin premix^d^	0.20	0.20	0.20	0.20	0.20	0.20
Mineral premix^b^	0.50	0.50	0.50	0.50	0.50	0.50
Choline chloride	0.20	0.20	0.20	0.20	0.20	0.20
Ca(H_2_PO_4_)_2_	2.00	2.00	2.00	2.00	2.00	2.00
BHT	0.20	0.20	0.20	0.20	0.20	0.20
Kelp powder	2.00	2.00	2.00	2.00	2.00	2.00
Total	100.00	100.00	100.00	100.00	100.00	100.00
Nutrient levels^e^						
Dry matter	88.67	92.61	90.96	90.81	91.97	91.47
Crude protein	41.85	42.47	42.23	42.20	41.48	42.20
Crude lipid	12.52	13.67	14.00	13.79	13.30	12.80

Abbreviations: ARA, arachidonic acid; ARA0.1, 0% ARA; ARA0.59, 0.5% ARA; ARA1.04, 1.0% ARA; ARA1.42, 1.5% ARA; ARA1.94, 2.0% ARA; ARA2.42, 2.5% ARA; DHA, docosahexaenoic acid; EPA, eicosapentaenoic acid.

^a^ARA enriched oil: ARA content, 350 mg/g; DHA content, 64.62 mg/g; EPA content, 0.20 mg/g.

^b^DHA enriched oil: DHA content, 331.12 mg/g; EPA content, 8.35 mg/g.

^c^EPA enriched oil: DHA content, 58.82 mg/g; EPA content, 231.13 mg/g.

^d^Mineral mixture and vitamin mixture were purchased from Ningbo Tech-Bank Feed Co. Ltd., China.

^e^Nutrient levels were measured values.

**Table 2:** Growth performance of *Acanthopagrus schlegelii* fed different experimental diets for 8 weeks.

Parameters	Experimental diets							ANOVA	Linear		Quadratic	
	ARA0.1	ARA0.59	ARA1.04	ARA1.42		ARA1.94	ARA2.42	*p* value	*p* value	*R* ^2^	*p* value	*R* ^2^
IW (g)^1^	0.97 ± 0.00	1.03 ± 0.04	1.03 ± 0.03	0.98 ± 0.00		0.99 ± 0.02	0.99 ± 0.02	0.290	—	—	—	—
WG (%)^2^	2906.08 ± 40.57^b^	3048.12 ± 38.02^bc^	3142.06 ± 57.31^cd^		3298.36 ± 18.53^d^	2883.03 ± 47.68^b^	2493.77 ± 50.25^a^	*p* ≤ 0.001	0.060	0.203	*p* ≤ 0.001	0.852
SGR (% day^−1^)^3^	5.96 ± 0.02^bc^	6.05 ± 0.02^cd^	6.10 ± 0.03^de^	6.19 ± 0.01^e^		5.93 ± 0.01^b^	5.82 ± 0.03^a^	*p* ≤ 0.001	0.109	0.153	*p* ≤ 0.001	0.790
SR (%)^4^	100.00 ± 0.00	97.33 ± 2.67	97.33 ± 1.33	97.33 ± 2.67		97.33 ± 2.67	96.00 ± 2.31	0.864	0.234	0.087	0.472	0.095

*Note:* Values are represented as the means of three replications. Means in the same row with different superscripts are significantly different (*p*  < 0.05).

Abbreviations: ARA, arachidonic acid; ARA0.1, 0% ARA; ARA0.59, 0.5% ARA; ARA1.04, 1.0% ARA; ARA1.42, 1.5% ARA; ARA1.94, 2.0% ARA; ARA2.42, 2.5% ARA; IW, initial weight; SGR, specific growth rate; SR, survival rate; WG, weight gain.

^1^IW: total initial weight/initial fish number.

^2^WG (%) = 100 × ((final body weight) − (initial body weight))/(initial body weight).

^3^SGR (% day^−1^) = 100 × (Ln (final body weight) − Ln (initial body weight))/56 days.

^4^SR (%) = 100 × (final number of fish)/(initial number of fish).

## Data Availability

The data used to support the findings of this study are available from the corresponding author upon request.

## Supporting Information

Supporting Information Table S1: Fatty acid composition (mg/g, dry) of the experimental diets. Table S2: Real-time quantitative PCR primers for lipid metabolism related genes of *Acanthopagrus schlegelii*. Table S3: Effects of dietary ARA on complete fatty acid compositions (mg/g, dry) of liver in *A. schlegelii*. Table S4: Effects of dietary ARA on complete fatty acid compositions (mg/g, dry) of muscle in *A. schlegelii*.
