# Supplementary material for: Evaluation of the Optimum Dietary Arachidonic Acid Level and Its Essentiality for Black Seabream (Acanthopagrus schlegelii): Based on Growth and Lipid Metabolism
Source: Aquac Nutr. 2024 Nov 14;2024:5589032. doi: 10.1155/2024/5589032 (PMC11581799; doi:10.1155/2024/5589032)
Supplement: Supporting Information — Table S1: Fatty acid composition (mg/g, dry) of the experimental diets. Table S2: Real-time quantitative PCR primers for lipid metabolism related genes of Acanthopagrus schlegelii. Table S3: Effects of dietary ARA on complete fatty acid compositions (mg/g, dry) of liver in A. schlegelii. Table S4: Effects of dietary ARA on complete fatty acid compositions (mg/g, dry) of muscle in A. schlegelii. [file 5589032.f1.docx]

**Supplementary Material**

Table S1: Fatty acid composition (mg/g, dry) of the experimental diets.

Table S2: Real-time quantitative PCR primers for lipid metabolism related genes of *Acanthopagrus schlegelii*.

Table S3: Effects of dietary ARA on complete fatty acid compositions (mg/g, dry) of liver in *Acanthopagrus schlegelii*.

Table S4: Effects of dietary ARA on complete fatty acid compositions (mg/g, dry) of muscle in *Acanthopagrus schlegelii*.

**Table S1**

Fatty acid compositions (mg/g, dry) of the experimental diets.

| Items | Experimental diets | | | | | |
| --- | --- | --- | --- | --- | --- | --- |
|  | ARA0.10 | ARA0.59 | ARA1.04 | ARA1.42 | ARA1.94 | ARA2.42 |
| 14:0 | 1.72 | 1.63 | 1.56 | 1.38 | 1.47 | 1.41 |
| 16:0 | 40.39 | 35.21 | 29.25 | 24.22 | 17.86 | 10.28 |
| 18:0 | 2.48 | 3.01 | 3.50 | 3.96 | 4.52 | 4.94 |
| 20:0 | 0.14 | 0.23 | 0.31 | 0.36 | 0.48 | 0.54 |
| ∑SFA^1^ | 44.73 | 40.08 | 34.61 | 29.92 | 24.32 | 17.17 |
| 16:1n | 2.59 | 2.56 | 2.48 | 2.38 | 2.57 | 2.56 |
| 18:1n-9 | 10.87 | 11.80 | 12.50 | 12.98 | 14.20 | 14.88 |
| 20:1n-9 | 0.45 | 0.47 | 0.54 | 0.56 | 0.61 | 0.60 |
| 22:1n-11 | 0.10 | 0.11 | 0.10 | 0.10 | 0.09 | 0.13 |
| ∑MUFA^2^ | 14.01 | 14.93 | 15.62 | 16.03 | 17.47 | 18.17 |
| 18:2n-6 | 4.12 | 4.34 | 4.53 | 4.66 | 5.15 | 5.32 |
| 18:3n-6 | 0.12 | 0.34 | 0.55 | 0.78 | 1.12 | 1.31 |
| 20:2n-6 | 0.11 | 0.12 | 0.17 | 0.21 | 0.28 | 0.31 |
| 20:4n-6 | 0.98 | 5.90 | 10.38 | 14.15 | 19.38 | 24.17 |
| 22:4n-6 | 0.00 | 0.00 | 0.31 | 0.46 | 0.42 | 0.45 |
| n-6PUFA^3^ | 5.33 | 10.71 | 15.95 | 20.25 | 26.34 | 31.56 |
| 18:3n-3 | 1.10 | 1.09 | 1.06 | 1.02 | 1.16 | 1.12 |
| 18:4n-3 | 0.61 | 0.61 | 0.59 | 0.53 | 0.65 | 0.64 |
| 20:4n-3 | 0.35 | 0.36 | 0.38 | 0.38 | 0.41 | 0.30 |
| 20:5n-3 | 10.64 | 10.53 | 10.38 | 9.64 | 10.95 | 10.98 |
| 22:5n-3 | 0.92 | 0.98 | 0.97 | 0.90 | 1.04 | 1.03 |
| 22:6n-3 | 8.75 | 9.02 | 9.21 | 9.02 | 9.72 | 10.32 |
| n-3PUFA^4^ | 22.37 | 22.59 | 22.58 | 21.48 | 23.93 | 24.39 |
| n-3LCPUFA^5^ | 20.66 | 20.89 | 20.94 | 19.93 | 22.11 | 22.62 |

**Notes:** ^1^ SFA, saturated fatty acids; ^2^ MUFA, monounsaturated fatty acids; ^3^ n-6 PUFA, n-6 polyunsaturated fatty acids; ^4^ n-3 PUFA, n-3 polyunsaturated fatty acids; ^5^ n-3LCPUFA, n-3 long chain polyunsaturated fatty acids. Values are presented as the means ± SEM of three replicates (n= 3).

**Table S2**

| Gene | Nucleotide sequence (from 5′ to 3′) | Size (bp) | Accession no.  or Publication | Functions |
| --- | --- | --- | --- | --- |
| *fas*^1^ | F: AAGAGCAGGGAGTGTTCGC  R: TGACGTGGTATTCAGCCGA | 213 | KX066240 | Lipogenesis pathway |
| *srebp1*^2^ | F: TGGGGGTAGGAGTGAGTAG  R: GTGAAGGGTCAGTGTTGGA | 247 | KX066235 | Lipogenesis pathway |
| *fabp*^3^ | F: AAGCCGACGGTAGCCATCA  R: TTGCCATCCCACTTCTGC | 191 | AF501322 | Lipogenesis pathway |
| *lpl*^4^ | F: CTGCTACTCCTCTGCCCA  R: ACATCCCTGTTACCGTCC | 204 | KX078571 | Lipolysis pathway |
| *cpt1a*^5^ | F: TGCTCCTACACACTATTCCCA  R: CATCTGCTGCTCTATCTCCCG | 203 | KX078572 | Lipolysis pathway |
| *hsl*^6^ | F: AGCAACTAAGCCCTCCCCATC  R: TCTTCACCCAGTCCGACACAC | 179 | KX066236 | Lipolysis pathway |
| *pparα*^7^ | F: ACGACGCTTTCCTCTTCCC  R: GCCTCCCCCTGGTTTATTC | 183 | KX066234 | Lipolysis pathway |
| *sirt1*^8^ | F: TGGATGAAACTGTAGGAACC  R: ACAACAATGGACTGGGAA | 238 | MN871952 | Metabolic sensor |
| *accα*^9^ | F: AGTAGCCTGATTCGTTGGT  R: AGTAGCCTGATTCGTTGGT | 154 | KX066238 | Lipolysis pathway |
| *atgl*^10^ | F: GCATCCAGTTCACCCTCAC  R: TTTGCCTCATCTTCATCGC | 241 | KX078570 | Lipolysis pathway |
| *β-actin*^11^ | F: ACCCAGATCATGTTCGAGACC  R: ATGAGGTAGTCTGTGAGGTCG | 212 | (Jiao et al., 2006) | Housekeeping gene |

Real-time quantitative PCR primers for lipid metabolism related genes of *Acanthopagrus schlegelii.*

**Notes:** ^1^ *fas*, fatty acid synthetase; ^2^ *srebp-1*, sterol regulatory element-binding protein-1; ^3^ *fadp*, fatty acid-binding protein; ^4^ *lpl*, lipoprotein lipase; ^5^ *cpt1a*, carnitine palmitoyltransferase 1a; ^6^ *hsl*, hormone-sensitive lipase; ^7^ *pparα*, peroxisome proliferators-activated receptor alpha; ^8^ *sirt1*, sirtuin type 1;^9^ *acca,* acetyl-CoA carboxylase alpha; ^10^ *atgl,* adipose triglyceride lipase.

**Table S3**

Effects of dietary ARA on complete fatty acid compositions (mg/g, dry) of liver in *Acanthopagrus schlegelii*.

| Items | ARA0.1 | ARA0.59 | ARA1.04 | ARA1.42 | ARA1.94 | ARA2.42 | ANOVA  *P*-value | ANOVA  F-value |
| --- | --- | --- | --- | --- | --- | --- | --- | --- |
| 14:0 | 5.43±0.46 | 4.64±0.39 | 4.59±0.12 | 4.85±0.31 | 5.65±0.11 | 5.16±0.04 | 2.35 | 0.11 |
| 16:0 | 63.86±0.57^b^ | 58.86±0.30^a^ | 58.83±0.16^a^ | 66.34±0.82^bc^ | 67.83±1.24^c^ | 56.95±0.70^a^ | 38.88 | 0.01 |
| 18:0 | 34.74±0.15^b^ | 29.70±1.38^a^ | 33.81±0.43^ab^ | 33.54±0.08^ab^ | 39.55±1.00^c^ | 33.92±1.37^ab^ | 12.02 | 0.00 |
| 20:0 | 0.91±0.10 | 0.87±0.09 | 0.91±0.03 | 1.07±0.01 | 1.11±0.04 | 0.87±0.03 | 2.90 | 0.06 |
| ∑SFA^1^ | 104.95±0.10^b^ | 94.07±1.80^a^ | 98.15±0.57^a^ | 105.80±1.05^b^ | 114.14±2.32^c^ | 95.13±0.40^a^ | 34.72 | 0.00 |
| 16:1n | 8.73±0.45 | 8.64±0.72 | 8.24±0.21 | 8.53±0.22 | 9.13±0.41 | 7.83±0.34 | 1.07 | 0.42 |
| 18:1n-9 | 91.42±0.33^c^ | 83.13±1.50^b^ | 83.31±0.55^b^ | 84.61±0.68^b^ | 89.82±0.70^c^ | 77.34±1.30^a^ | 29.34 | 0.00 |
| 20:1n-9 | 2.87±0.14 | 2.85±0.10 | 2.58±0.09 | 2.74±0.13 | 2.48±0.13 | 2.16±0.28 | 2.96 | 0.06 |
| 22:1n-11 | 1.10±0.13 | 0.87±0.06 | 0.90±0.06 | 1.00±0.05 | 0.90±0.05 | 0.77±0.10 | 2.01 | 0.15 |
| ∑MUFA^2^ | 104.11±0.60^c^ | 95.48±1.14^b^ | 95.04±0.41^b^ | 96.88±1.00^b^ | 102.34±0.88^c^ | 88.10±1.03^a^ | 42.22 | 0.00 |
| 18:2n-6 | 7.27±0.41^a^ | 8.09±0.30^ab^ | 8.33±0.02^bc^ | 8.01±0.05^ab^ | 9.26±0.05^c^ | 9.23±0.18^c^ | 12.04 | 0.00 |
| 18:3n-6 | 1.55±0.05^a^ | 2.12±0.21^ab^ | 2.72±0.05^bc^ | 3.05±0.14^c^ | 4.72±0.19^d^ | 6.18±0.04^e^ | 175.39 | 0.00 |
| 20:2n-6 | 0.98±0.14^a^ | 1.06±0.04^ab^ | 1.19±0.02^ab^ | 1.17±0.01^ab^ | 1.33±0.06^bc^ | 1.55±0.06^c^ | 8.89 | 0.00 |
| 20:4n-6 | 7.42±0.54^a^ | 16.66±0.21^b^ | 28.32±0.20^c^ | 33.90±0.62^d^ | 49.62±0.65^e^ | 57.05±0.94^f^ | 1039.40 | 0.00 |
| n-6PUFA^3^ | 17.21±1.09^a^ | 27.93±0.41^b^ | 40.55±0.18^c^ | 46.13±0.73^d^ | 64.93±0.82^e^ | 74.01±0.89^f^ | 818.66 | 0.00 |
| 18:3n-3 | 1.43±0.01^c^ | 1.22±0.07^abc^ | 1.31±0.08^bc^ | 1.01±0.03^a^ | 1.11±0.06^ab^ | 1.44±0.07^c^ | 8.32 | 0.01 |
| 18:4n-3 | 0.72±0.01^ab^ | 0.59±0.04^a^ | 0.70±0.01^ab^ | 0.57±0.03^a^ | 0.73±0.06^ab^ | 0.91±0.10^b^ | 5.31 | 0.01 |
| 20:4n-3 | 2.23±0.12^c^ | 2.02±0.06^abc^ | 1.93±0.04^ab^ | 1.76±0.04^a^ | 2.1±0.05^bc^ | 2.58±0.02^d^ | 19.95 | 0.00 |
| 20:5n-3 | 20.23±0.22^d^ | 18.38±0.36^c^ | 17.77±0.04^c^ | 15.74±0.05^b^ | 15.82±0.04^b^ | 14.64±0.19^a^ | 116.10 | 0.00 |
| 22:5n-3 | 17.56±0.30^c^ | 15.83±0.18^b^ | 15.93±0.25^b^ | 13.42±0.17^a^ | 15.17±0.60^b^ | 15.51±0.06^b^ | 18.27 | 0.00 |
| 22:6n-3 | 34.52±0.41^d^ | 35.24±0.57^d^ | 30.93±0.38^c^ | 24.09±0.25^b^ | 23.80±0.05^b^ | 21.25±0.35a | 263.79 | 0.00 |
| n-3PUFA^4^ | 76.69±0.49^e^ | 73.28±0.58^d^ | 69.11±0.01^c^ | 56.17±0.07^a^ | 58.31±0.13^b^ | 55.94±0.12^a^ | 833.61 | 0.00 |
| n-3LCPUFA^5^ | 74.54±0.48^e^ | 71.47±0.48^d^ | 66.56±0.48^c^ | 55.01±0.42^ab^ | 56.90±0.57^b^ | 53.97±0.34^a^ | 366.65 | 0.00 |

**Notes:** Values are represented as the means of three replications. Means in the same row with different superscripts are significantly different (*P* < 0.05).

^1^ SFA, saturated fatty acids; ^2^ MUFA, monounsaturated fatty acids; ^3^ n-6 PUFA, n-6 polyunsaturated fatty acids; ^4^ n-3 PUFA, n-3 polyunsaturated fatty acids; ^5^ n-3 LCPUFA, n-3 long chain polyunsaturated fatty acids.

**Table S4**

Effects of dietary ARA on complete fatty acid compositions (mg/g, dry) of muscle in *Acanthopagrus schlegelii*.

| Items | ARA0.1 | ARA0.59 | ARA1.04 | ARA1.42 | ARA1.94 | ARA2.42 | ANOVA  *P*-value | ANOVA  *F*-value |
| --- | --- | --- | --- | --- | --- | --- | --- | --- |
| 14:0 | 0.81±0.02 | 0.85±0.11 | 0.86±0.04 | 0.84±0.07 | 0.75±0.04 | 0.94±0.04 | 2.29 | 0.12 |
| 16:0 | 11.88±0.44^ab^ | 11.16±0.1^a^ | 12.24±0.36^b^ | 11.85±0.15^ab^ | 11.67±0.11^ab^ | 11.88±0.06^ab^ | 4.26 | 0.02 |
| 18:0 | 2.76±0.08^a^ | 3.06±0.02^b^ | 3.25±0.01^c^ | 3.53±0.04^d^ | 4.08±0.02^e^ | 4.32±0.05^f^ | 186.91 | 0.00 |
| 20:0 | 0.07±0.00^a^ | 0.10±0.01^b^ | 0.13±0.01^bc^ | 0.15±0.01^cd^ | 0.16±0.01^d^ | 0.22±0.02^e^ | 54.59 | 0.06 |
| ∑SFA^1^ | 15.52±0.07^a^ | 15.17±0.08^a^ | 16.11±0.13^b^ | 16.37±0.17^bc^ | 16.66±0.13^c^ | 17.37±0.09^d^ | 46.53 | 0.00 |
| 16:1n | 1.62±0.01^a^ | 1.72±0.05^ab^ | 1.84±0.03^b^ | 1.82±0.01^b^ | 1.71±0.07^ab^ | 1.82±0.02^b^ | 5.53 | 0.01 |
| 18:1n-9 | 10.94±0.47^a^ | 10.19±0.12^a^ | 11.45±0.05^ab^ | 12.24±0.18^b^ | 10.68±0.4^a^ | 12.46±0.26^b^ | 9.69 | 0.00 |
| 20:1n-9 | 0.19±0.02^a^ | 0.23±0.01^ab^ | 0.25±0.01^abc^ | 0.28±0.02^bc^ | 0.24±0.01^abc^ | 0.31±0.01^c^ | 6.92 | 0.06 |
| 22:1n-11 | 0.13±0.01 | 0.13±0.01 | 0.14±0.01 | 0.12±0.01 | 0.13±0.00 | 0.14±0.01 | 1.34 | 0.31 |
| ∑MUFA^2^ | 12.88±0.47^ab^ | 12.27±0.16^a^ | 13.69±0.06^bc^ | 14.45±0.19^c^ | 12.77±0.35^ab^ | 14.74±0.29^a^ | 12.05 | 0.00 |
| 18:2n-6 | 1.75±0.03^a^ | 2.15±0.00^b^ | 2.55±0.01^c^ | 2.50±0.06^c^ | 2.41±0.10^c^ | 3.09±0.11^d^ | 44.29 | 0.00 |
| 18:3n-6 | 0.17±0.01^a^ | 0.28±0.02^b^ | 1.16±0.01^c^ | 0.52±0.02^cd^ | 0.59±0.01^d^ | 0.88±0.02^f^ | 204.60 | 0.00 |
| 20:2n-6 | 0.10±0.01^a^ | 0.11±0.00^ab^ | 0.13±0.00^bc^ | 0.14±0.01^c^ | 0.16±0.01^c^ | 0.21±0.00^d^ | 50.54 | 0.00 |
| 20:4n-6 | 1.03±0.04^a^ | 3.74±0.18^b^ | 6.78±0.06^c^ | 8.78±0.09^d^ | 11.55±0.45^e^ | 14.72±0.38^f^ | 386.69 | 0.00 |
| n-6 PUFA^3^ | 1.44±0.06^a^ | 4.52±0.17^b^ | 8.8±0.59^c^ | 10.38±0.05^d^ | 13.46±0.5^e^ | 17.46±0.45^f^ | 244.96 | 0.00 |
| 18:3n-3 | 0.34±0.01^a^ | 0.43±0.01^bc^ | 0.48±0.00^c^ | 0.42±0.03^bc^ | 0.41±0.01^b^ | 0.48±0.01^c^ | 13.02 | 0.00 |
| 18:4n-3 | 0.18±0.01^a^ | 0.22±0.01^b^ | 0.27±0.01^cd^ | 0.24±0.01^bc^ | 0.20±0.01^ab^ | 0.29±0.01^d^ | 18.57 | 0.00 |
| 20:4n-3 | 0.30±0.01 | 0.30±0.02 | 0.31±0.02 | 0.29±0.02 | 0.25±0.01 | 0.32±0.01 | 2.21 | 0.12 |
| 20:5n-3 | 5.57±0.17^d^ | 5.18±0.00^cd^ | 5.14±0.18^cd^ | 4.73±0.09^bc^ | 4.35±0.03^ab^ | 4.23±0.10^a^ | 20.42 | 0.00 |
| 22:5n-3 | 1.61±0.00^b^ | 1.55±0.01^b^ | 1.52±0.01^b^ | 1.39±0.01^a^ | 1.39±0.06^a^ | 1.35±0.01^a^ | 17.40 | 0.00 |
| 22:6n-3 | 9.24±0.05^d^ | 8.19±0.16^c^ | 7.26±0.11^b^ | 6.75±0.11^b^ | 5.20±0.13^a^ | 4.87±0.20^a^ | 147.91 | 0.00 |
| n-3 PUFA^4^ | 17.24±0.13^e^ | 15.88±0.19^d^ | 14.98±0.13^c^ | 13.8±0.24^b^ | 11.81±0.17^a^ | 11.53±0.24^a^ | 142.89 | 0.00 |
| n-3 LC-PUFA^5^ | 16.55±0.19^e^ | 15.23±0.22^d^ | 14.30±0.17^c^ | 13.25±0.26^b^ | 11.19±0.17^a^ | 10.73±0.08^a^ | 142.89 | 0.00 |

**Notes:** Values are represented as the means of three replications. Means in the same row with different superscripts are significantly different (*P* < 0.05).

^1^ SFA, saturated fatty acids; ^2^ MUFA, monounsaturated fatty acids; ^3^ n-6 PUFA, n-6 polyunsaturated fatty acids; ^4^ n-3 PUFA, n-3 polyunsaturated fatty acids; ^5^ n-3LCPUFA, n-3 long chain polyunsaturated fatty acids.
